# Supplementary material for: Localized Corrosion of Stainless Steel Triggered by Typical Inclusions in NaCl Solution: Oxy-Sulfide and MnS
Source: Materials (Basel). 2023 Jun 12;16(12):4323. doi: 10.3390/ma16124323 (PMC10302553; doi:10.3390/ma16124323)
Supplement: Supplementary file 1 [file materials-16-04323-s001.zip › materials-2397245-supplementary.pdf]

Localized corrosion of stainless steel triggered by typical inclusions in NaCl solution: MnS and mixed oxide/sulfide inclusion

Dan Li, Hui Zhi, Zhaogui Lai and Ying Jin *

National Center for Materials Service Safety, University of Science and Technology Beijing, Beijing 100083, China; lidan9203@126.com (D.L.); neu3dmaker@163.com (H.Z.); laizhaogui@ustb.edu.cn (Z.L.)
* Correspondence: yjin@ustb.edu.cn

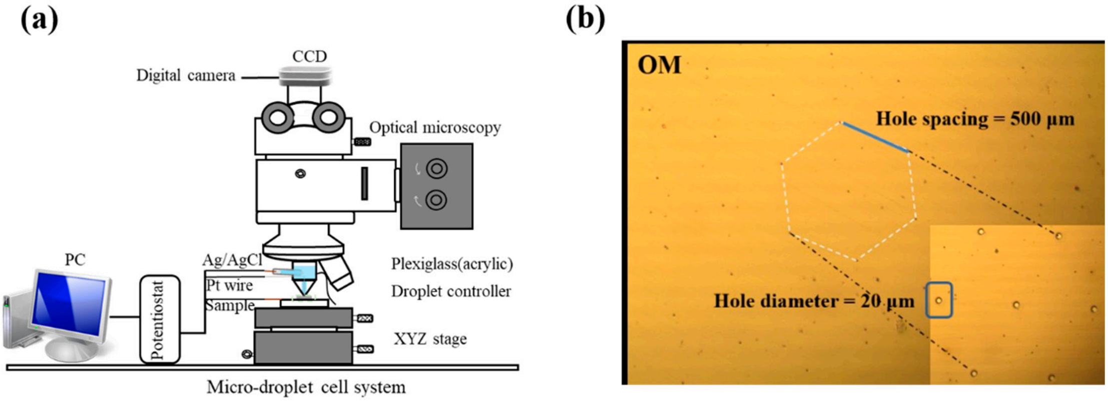

Figure S1. (**a**) Schematic diagram of home-made microelectrochemical test system, (**b**) sample coated with photoresist with a thickness of about 2.5 μm.

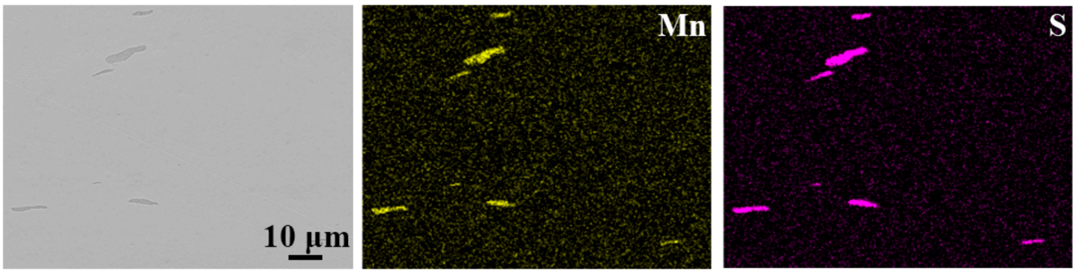

Figure S2. Rod-shaped MnS inclusions formed in the samples.

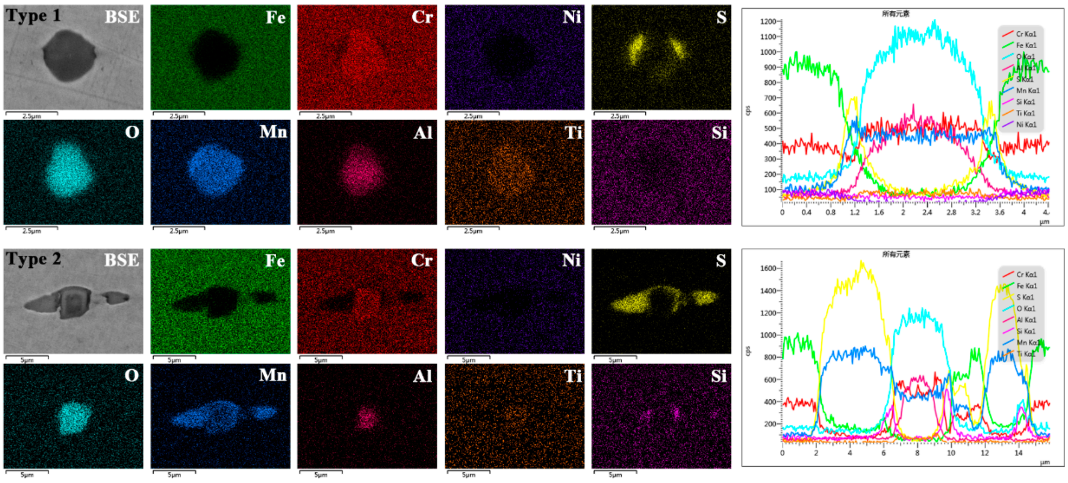

Figure S3. EDS results of two types of oxy-sulfide inclusions. Type 1: polygonal oxide wrapped by rounded sulfide; Type 2: polygonal oxide embedded in rod-shaped sulfide.

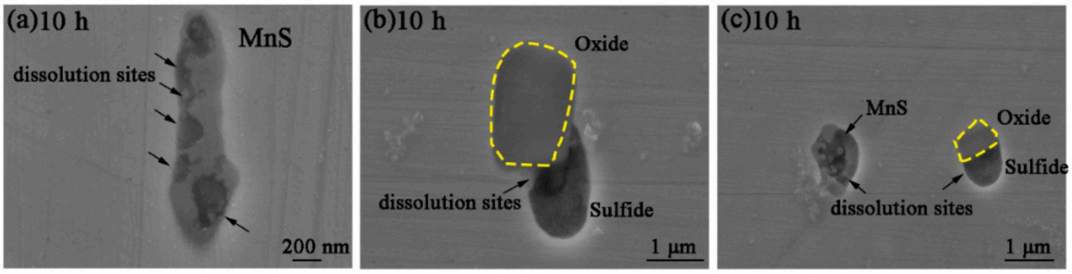

Figure S4. (**a**) The MnS always starts to dissolve from the edge areas; (**b**) The sulfide parts in mixed oxide/sulfide inclusions tend to dissolve preferentially from the boundary between the oxide parts and sulfide parts; (**c**) Corrosion morphology of two inclusions in the same field of view
